# Coordinate Regulation of G Protein Signaling via Dynamic Interactions of Receptor and GAP

**DOI:** 10.1371/journal.pcbi.1000148

**Published:** 2008-08-15

**Authors:** Marc Turcotte, Wei Tang, Elliott M. Ross

**Affiliations:** 1 Department of Pharmacology, University of Texas Southwestern Medical Center, Dallas, Texas, United States of America; 2 Department of Cell Biology, University of Texas Southwestern Medical Center, Dallas, Texas, United States of America; University of Montana, United States of America

## Abstract

Signal output from receptor–G-protein–effector modules is a dynamic function of the nucleotide exchange activity of the receptor, the GTPase-accelerating activity of GTPase-activating proteins (GAPs), and their interactions. GAPs may inhibit steady-state signaling but may also accelerate deactivation upon removal of stimulus without significantly inhibiting output when the receptor is active. Further, some effectors (e.g., phospholipase C-β) are themselves GAPs, and it is unclear how such effectors can be stimulated by G proteins at the same time as they accelerate G protein deactivation. The multiple combinations of protein–protein associations and interacting regulatory effects that allow such complex behaviors in this system do not permit the usual simplifying assumptions of traditional enzyme kinetics and are uniquely subject to systems-level analysis. We developed a kinetic model for G protein signaling that permits analysis of both interactive and independent G protein binding and regulation by receptor and GAP. We evaluated parameters of the model (all forward and reverse rate constants) by global least-squares fitting to a diverse set of steady-state GTPase measurements in an m1 muscarinic receptor–G_q_–phospholipase C-β1 module in which GTPase activities were varied by ∼10^4^-fold. We provide multiple tests to validate the fitted parameter set, which is consistent with results from the few previous pre-steady-state kinetic measurements. Results indicate that (1) GAP potentiates the GDP/GTP exchange activity of the receptor, an activity never before reported; (2) exchange activity of the receptor is biased toward replacement of GDP by GTP; (3) receptor and GAP bind G protein with negative cooperativity when G protein is bound to either GTP or GDP, promoting rapid GAP binding and dissociation; (4) GAP indirectly stabilizes the continuous binding of receptor to G protein during steady-state GTPase hydrolysis, thus further enhancing receptor activity; and (5) receptor accelerates GDP/GTP exchange primarily by opening an otherwise closed nucleotide binding site on the G protein but has minimal effect on affinity (*K*_assoc_ = *k*_assoc_/*k*_dissoc_) of G protein for nucleotide. Model-based simulation explains how GAP activity can accelerate deactivation >10-fold upon removal of agonist but still allow high signal output while the receptor is active. Analysis of GTPase flux through distinct reaction pathways and consequent accumulation of specific GTPase cycle intermediates indicate that, in the presence of a GAP, the receptor remains bound to G protein throughout the GTPase cycle and that GAP binds primarily during the GTP-bound phase. The analysis explains these behaviors and relates them to the specific regulatory phenomena described above. The work also demonstrates the applicability of appropriately data-constrained system-level analysis to signaling networks of this scale.

## Author Summary

Throughout the eukaryotes, G proteins convey information from receptors for diverse stimuli—neurotransmitters, hormones, light, odors, and pheromones—to intracellular regulatory proteins collectively known as effectors. G proteins function by transiting a dynamic cycle of activation and deactivation. Receptors accelerate activation, which allows G proteins to regulate effectors, and receptors thus increase signal output. GTPase-activating proteins, GAPs, accelerate deactivation. GAPs can thus attenuate signaling, but GAPs can also accelerate signal termination when stimulus is removed without inhibiting signal output while stimulus is present. Surprisingly, some effectors are also GAPs for the G proteins that activate them, essentially turning off their activator. We developed a mathematical model that describes control of G protein signaling by receptor and GAP and used experimental data to determine its important parameters. We show that GAPs actually potentiate G protein activation by receptor, a previously unsuspected effect. Further, GAPs indirectly stabilize receptor–G protein binding during stimulation, which we had previously predicted based on inconsistencies among other experimental results. The present results elucidate how GAPs can independently control signaling kinetics and amplitude and thus clarify how effectors can both respond to G proteins and act as G protein GAPs.

## Introduction

G protein-mediated signaling modules display a variety of dynamic input-output behaviors despite their use of a single, relatively simple biochemical mechanism. Signal amplification, the ratio of effector proteins activated to agonist-bound receptors, can vary from unity to hundreds. Activating ligands may bind receptors with affinities ranging from picomolar through millimolar. GAPs, which can accelerate hydrolysis of bound GTP over 2000-fold, can accelerate both activation and deactivation in cells with variable inhibitory effect [1]. Activation and deactivation rates upon addition and removal of agonist can thus range from ∼10 ms to minutes.

Heterotrimeric G proteins convey signals by traversing a cycle of GTP binding and hydrolysis: the GTP bound state of the Gα subunit is active and deactivation is caused by hydrolysis of bound GTP to GDP [2]. The rates of activation and deactivation, and consequent effects on signal output at steady state, are regulated by interactions of the Gα subunit with receptors [3], Gβγ subunits [4], GTPase-activating proteins (GAPs) [1] and multiple other proteins [5]. The net effect of these inputs depends on the identities of the individual proteins, their concentrations and their own regulatory controls. Regulatory inputs to G protein modules are interactive, and it has been difficult to establish quantitative understanding of how they cooperate to control signal output. While some signals, particularly G protein-gated channels, can be monitored accurately in cells in real time, it has been harder to quantitate the intermediary reactions of the GTPase cycle and protein–protein binding or dissociation. Recently developed optical sensors are promising [6]–[10] but still do not provide complete or simultaneous coverage of multiple events and often do not provide absolute (i.e., molar) data. Conversely, quantitative biochemical assays using in vitro reconstituted systems have provided absolute biochemical data [11],[12] but have not adequately described the simultaneous regulatory interactions that are so important. Consequently, quantitative understanding of the dynamic behavior of an intact G protein module remains elusive.

Computational modeling is used frequently to clarify mechanistic thinking about complex biochemical systems, including G protein signaling. Quantitative models can potentially combine information on individual reactions to simulate the behavior of a complex system, or use system-level data to test the validity of a proposed mechanism. The work of Linderman and colleagues, for example, has provided consistent examples of these approaches to G protein signaling [13]–[16]. The G protein-mediated yeast pheromone response has also been the focus of significant modeling efforts because of its presumed paucity of off-pathway inputs [17]–[19]. In at least one case, the failure of a simple model of this pathway motivated discovery of a novel mechanism for feedback regulation and subsequent refinement of the model [17]. However, modeling of G protein modules has often been descriptive, with parameters arbitrarily chosen for a few reactions such that model output mimics an experimental result. Alternatively, the inner workings of the G protein module itself have been condensed into an arbitrary function of agonist concentration and receptor regulation to allow analysis of a downstream event such as Ca^2+^ release or protein phosphorylation or, even more distal, transcription.

A major problem in developing quantitative models of G protein modules has been accurate assignment of parameters to the many processes that are known to occur. These include both the GTPase cycle reactions and the multiple protein-protein interactions that govern these reactions. This problem is significant because local protein concentrations at the plasma membrane and the regulated association of these proteins are both unknown, either for resting cells or during dynamic signaling. In this study, we have used purified proteins, heterotrimeric G_q_, m1 muscarinic acetylcholine receptors and phospholipase C-β1, reconstituted at uniform and controllable concentrations into unilamellar phospholipid vesicles, to overcome this first limitation. We estimated formation of multi-protein complexes according to their individual activities.

The second major problem in modeling signaling through G protein modules is the difficulty in assigning correct, or even plausible, values of rate or equilibrium constants for the reactions included in the model. Despite their apparently small size, an informative model of a single G protein module will contain multiple parameters that are not readily accessible from individual measurements. These parameters may vary widely among different modules (receptors, G proteins, GAPs), which prohibits most literature-mining approaches. If all or most of the relevant parameters are not individually available for the module of interest, then an adequately large and diverse dataset must be produced to allow parameters to be fit to the data.

Last, even with a presumably adequate dataset, the numerical fitting process that extracts values for the parameters and subsequent validation of the fit are both central problems in modeling signaling systems. We have adapted and extended several approaches to deal with the difficulty of fitting a model with a fairly large number of parameters using a modest amount of data. We present a modestly complex model of signal output in a G protein model that contains many of the salient regulatory interactions that characterize G protein signaling. We used steady-state GTPase data to support a Metropolis-Monte Carlo fitting strategy, and argue that most parameters are reasonably assigned, with statistical data to help qualify fits for individual parameters.

The resultant parameter set shows that receptor accelerates both GDP dissociation and GTP binding, and that GAPs potentiate the receptor's nucleotide exchange catalyst activity. Further, the model argues strongly that GAP activity indirectly favors continued binding of receptor to G protein throughout the GTPase cycle, thus further potentiating the receptor's activity. Such indirect stabilization of receptor-G protein binding, referred to as kinetic scaffolding to distinguish it from direct interaction, was suggested as a mechanism for how a GAP can accelerate deactivation upon removal of agonist without substantially inhibiting signaling [1],[11],[16],[20]. Model-based simulation of signal output describes how GAPs combine these mechanisms to independently control signal amplitude and kinetics.

## Results

### Fitting the Model Using Steady-State Kinetic Data

**Figure 1:**
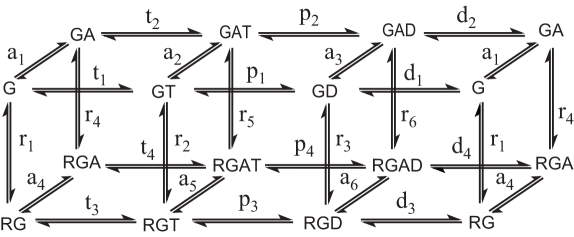
Thermodynamically complete model of the GTPase reaction catalyzed by G protein (G) and regulated by the reversible binding of receptor (R) and/or GAP (A). Reactions and related rate constants are named “a”, “r”, “t” and “d” for association of GAP, receptor, GTP (T) and GDP (D); “p” denotes GTP hydrolysis. The numeric subscript in the figure specifies the reaction shown. All reactions are reversible. (A second subscript in Table 1 and Figure S1 specifies association (“1”) or dissociation (“2”). For hydrolysis rate constants (“p”), “1” indicates GTP hydrolysis and “2” indicates synthesis.)

The biochemical model of the GTPase catalytic cycle (Figure 1) includes GTP binding, hydrolysis of bound GTP and simultaneous release of inorganic phosphate (Pi), and the dissociation of GDP. At each stage of the reaction, G protein is allowed to bind agonist-liganded receptor, GAP or both. Receptor is assumed to be agonist bound and active at all times; agonist-stimulated GTPase data were obtained in the presence of saturating carbamylcholine (1 mM). Possible dissociation of Gβγ from Gα and protein oligomerization were not included (see Discussion). All reactions were considered to be reversible to allow imposition of path-independence constraints on closed reaction loops during the fitting process (see below). For the same reason, even presumably unlikely reaction paths were retained to create symmetry in the reaction map. For calculation of G protein activation (see below), all GTP-bound species were considered to be equally active, and fractional activation was calculated as the fraction of all species that contain bound GTP.

**Figure 2:**
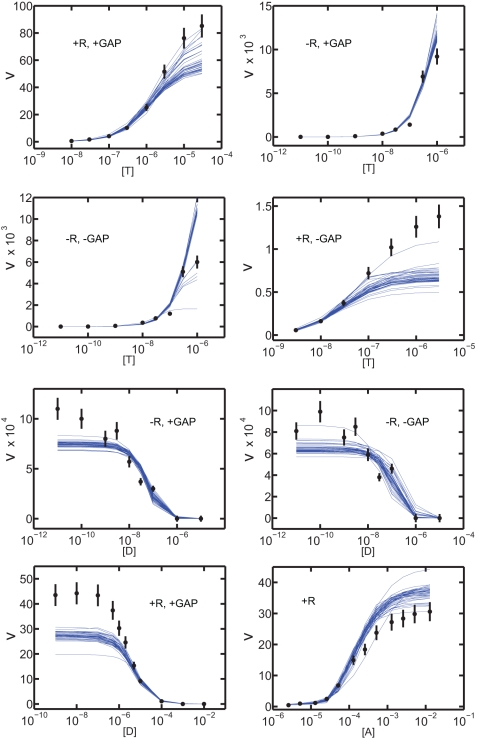
Agreement of simulations (blue) with GTPase rate data (black). Steady-state GTPase activity (moles GTP hydrolyzed/min/mole G_q_; ±SD) was measured in the presence or absence of 20 nM PLC-β1 (“+GAP) and/or 0.1 mM carbachol (“+R”) at varied molar concentrations of GTP, GDP or phospholipase (Table S1 for details). The family of simulations was generated using 41 sets of rate constants obtained from individual fits to the data. Note different scales on the V axes. Values of *K*_m_ for GTP, and its regulation by receptor and GAP, and for the EC_50_ for PLC-β1 are consistent with previously published results [11].

The kinetic model for G protein signaling (Figure 1) includes 48 parameters, first- and second-order rate constants, only a few of which have been determined directly. We therefore fit all the parameters to a relatively large and diverse set of steady-state GTPase rates determined in a purified and reconstituted system in which protein concentrations were known and where data could be obtained over a wide dynamic range. Data for fitting came from 8 scans of GTPase activity as the concentration of one assay component, GTP, GDP or GAP, was varied from zero to saturation in the presence or absence of saturating agonist (Table S1; Figure 2). Data were fit simultaneously to minimize the cost function, defined as the sum of the squares of deviations between experimental data and data predicted by the model (Materials and Methods). Values for the 48 kinetic parameters were adjusted simultaneously by constrained simulated annealing to best match all available data while satisfying thermodynamic constraints (path independence, i.e. cyclicΔ*G* = 0, for all potential cycles; and net Δ*G*_hydrol_ for GTP [21]). The progress of cost minimization for a typical fitting run is shown in Figure S2. The cost function is initially quite high (off-scale in Figure S2) and decreases rapidly. The initial decrease is followed by relatively quick adjustments of the parameters interspersed with long metastable stages, reflecting occasional escape of the Monte Carlo search from local minima in the cost manifold. Improvement in the fit is negligible past a few thousand iterations. To further test the adequacy of the Monte Carlo search, it was repeated with thermodynamic constraints applied as a quantitative penalty for nonconformance in the cost function rather than as an absolute constraint (Materials and Methods) (Figure S3). In this case, initial convergence was slower, but subsequent enforcement of strict thermodynamic constraints decreased the value of the cost function to a level similar to that achieved if thermodynamic constraints are applied throughout the fitting process. Because this more ergodic search method did not lead to lower values of the cost function, it is likely that imposing path-independence constraints initially does not seriously limit the ergodicity of the fitting process.

The initial test of such a modeling process is the ability of the model to simulate experimental data using the parameter set determined by fitting (Figure 2). Simulations based on the model and parameters derived from 41 fitting runs (Table S2) approximated the experimental data well over a 10^5^-fold range of GTPase activities and a wide variety of experimental conditions. Values of *K*_m_ for GTP, *K*_i_ for GDP and EC_50_ for the GAP activity of PLC-β1 were all matched closely in each experiment. Relative increases and decreases in activity were also simulated well, as were curve shape and steepness. The largest recurrent discrepancy between data and prediction was in the absolute value of the maximal activity. Disagreement was negligible in some experiments, but was significant in others. In part, this reflects real difficulty in fitting such a diverse dataset, but it also arises from variation in specific activity among the experiments. The data were obtained using several preparations of m1AChR-G_q_ vesicles that varied in maximum specific activities, with standard deviation of ∼40% among 13 batches of vesicles prepared during the study. Variation between fits and data in Figure 2 are within this margin.

**Figure 3:**
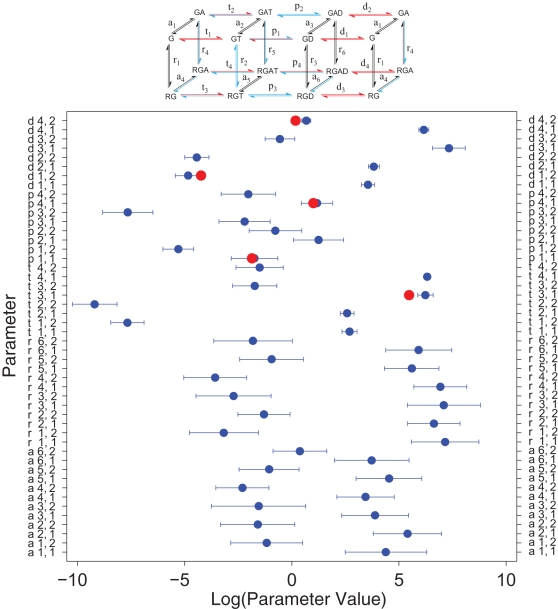
Rate constants for the G_q_-catalyzed GTPase cycle obtained by fitting steady-state kinetic data. Points show log-average values of parameters (±SD; Table S2) from 41 stochastic fitting searches performed as described in the Materials and Methods. Reactions are numbered and defined in Figure 1 and Figure S1. The letter and first number define the reaction and the second number defines forward (1) or reverse (2) rate constants. Red points are values determined previously by direct pre-steady-state measurements [12],[55]. The reaction scheme at the top color-codes rate constants according to the relative sizes of their confidence limits (Table S2): red, <10×; blue, <20×; black, >20×.

The values of the rate constants obtained by fitting the steady-state rates also compare well with those few that have previously been determined directly in pre-steady-state kinetic measurements [12] (Figure 3 **)**. For five reactions, nucleotide association and dissociation and GTP hydrolysis, agreement was within a factor of 4. The direct determinations were performed with different preparations of vesicles and by different investigators. Agreement is thus even more striking. Importantly, the pre-steady-state kinetic data were not used in the present fit. The rate constants obtained here also compare well with predictions from data obtained in non-identical preparations (detergent-solubilized proteins, free Gα_q_ subunits, etc.) [11],[12],[22],[23].

Fitting is a stochastic process that, upon repeat, converges to different minima of comparable cost in a complex manifold. For these datasets, multiple fitting runs yielded a family of parameter sets with cost functions in the range 650–800 (not shown). The extent of variation among repeated fits reflects the size of the error on each parameter (Figure 3). For some of the parameters, reproducibility was excellent, but for others error was large. Error may reflect the absence of necessary data or experimental error, but an additional difficulty in fitting some parameters arises from the structure of the model. To allow imposition of path-independence constraints, the model contains all possible interactions of proteins and nucleotides, including species that are quantitatively negligible and reactions that do not contribute detectably to flux through the GTPase cycle. Thus, some individual rate constants cannot be fit well, and some pairs of forward and reverse rate constants that describe rapid equilibria are poorly fit because the data only constrain their ratios.

**Figure 4:**
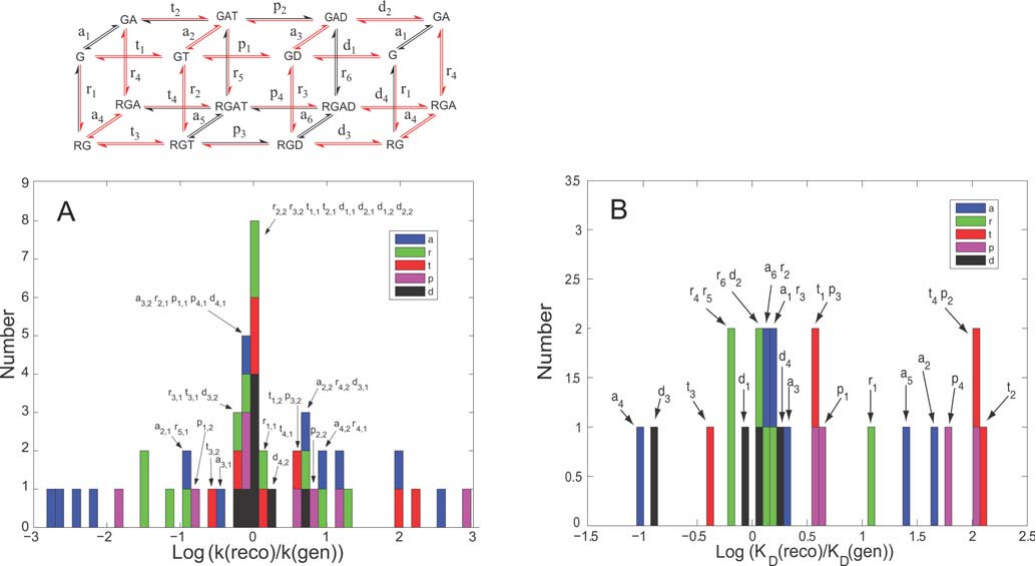
Reconstruction of model parameters by fitting synthetic data. Synthetic data similar to those of Figure 2 were generated by simulation and imposition of Gaussian noise. The synthetic data were then used for fitting to create a new best-fit parameter set. The histograms show the ratios of reconstructed parameters to the parameters used to generate the synthetic data. Only one fitting run was used to produce these values. (A) Rate constants. (B) Equilibrium constants. The reaction scheme at the top color codes rate constants in red if they were re-fit to within 10-fold of their original values.

To evaluate possible sources of errors associated with some of the parameters, we repeated the fitting process with synthetic data and asked whether the fitting process could accurately return the parameters used in the synthesis. Simulated data equivalent to the original experimental data were generated using the model and a chosen parameter set. To simulate experimental noise, Gaussian errors (standard deviation/mean = 10%) were convoluted with the predictions. The parameter set returned in this process simulated the synthetic data extremely well, and did not show the significant errors in maximal velocity observed when the real data were fitted (not shown). The parameter set obtained by fitting synthetic data was then compared with the set used in its generation (Figure 4A). The histogram shows that 32 of the 48 constants were fit to within 10-fold of the generating value, with 19 within 2-fold. Examination of the outliers indicates that they describe reactions that either are not appreciably populated or are much faster than the reaction that they precede, and therefore could not be constrained. The fitting process is thus adequate to determine most parameters well, and those that are not well fit do not contribute appreciably to overall flux through the GTPase cycle. To see whether rapid equilibria contribute to error in evaluating individual kinetic constants, we also compared the fitted equilibrium constants for each reaction (i.e., the ratios of forward and reverse rate constants) with the values used to generate the synthetic data (Figure 4B). Deviations from the generating values were fewer and smaller, indicating that equilibrium constants were constrained by the thermodynamic relationships used to construct the model. The quality of the fit was further assessed by thermal ensemble analysis [24] (Text S2). The analysis consists of generating statistically equivalent fits to the data and measures the extent to which parameters are coupled (Text S2). We found lack of generalized mixing suggesting (1) a reasonable match between the model and the underlying phenomena, (2) the absence of severe over- or under-parameterization, and (3) the availability of sufficient data for accurate determination of many of the parameters.

### Cooperative Interactions of G Protein, Receptor, and GAP

The parameter set shown in Figure 3 and Table S2 provides the first reasonably complete set of experimentally determined rate constants for a G protein signaling module, and thus provides insights into regulatory interactions that were not previously accessible. While the parameters are interpretable only to within the errors of the fit, several novel observations stand out at this level.

First, examination of the rates of nucleotide binding and release indicate that the salient function of receptor is to open an otherwise inaccessible (“closed”) nucleotide binding site on G_q_ to permit GDP/GTP exchange. In addition to accelerating GDP dissociation, receptor also markedly accelerates both GDP and GTP association (Table 1). Receptor thus promotes GDP/GTP exchange by two distinct mechanisms. It accelerates GDP dissociation over 10^4^-fold and GTP association more than 10^3^-fold. Receptors have been thought to act by binding G protein negatively cooperatively with respect to nucleotides; i.e., that receptor decreases affinity for GDP by increasing the dissociation rate (*K*_assoc_ = *k*_assoc_/*k*_diss_). In the case of the M1 muscarinic receptor and G_q_, the decrease in affinity for GDP (∼3-fold) is dwarfed by acceleration of GDP dissociation (∼20,000-fold; because GDP binding to the open site is also fast).

**Table 1:** Effect of receptor on nucleotide exchange kinetics and equilibria

Reaction	*k*_assoc_ (M^−1^·s^−1^)	+Rec/–Rec	*k*_dissoc_ (s^−1^)	+Rec/–Rec	*K*_eq_ (M^−1^)	+Rec/–Rec
t1	5.0×10^2^		2.2×10^−8^		2.3×10^10^	
t2 (+A)	3.8×10^2^		6.4×10^−10^		5.9×10^11^	
t3(+R)	1.7×10^6^	3.4×10^3^	1.9×10^−2^	8.6×10^5^	8.9×10^7^	3.9×10^−3^
t4(+A,+R)	2.1×10^6^	5.5×10^3^	3.2×10^−2^	5.0×10^7^	6.6×10^7^	1.1×10^−4^
d1	3.6×10^3^		1.5×10^−5^		2.4×10^8^	
d2(+A)	6.8×10^3^		3.7×10^−5^		1.8×10^8^	
d3(+R)	2.1×10^7^	5.8×10^3^	2.8×10^−1^	1.9×10^4^	7.5×10^7^	3.1×10^−1^
d4(+A,+R)	1.5×10^6^	2.2×10^2^	4.7	1.3×10^5^	3.2×10^5^	1.7×10^−3^

Rate constants are log average values from Table S2. “+Rec/–Rec” indicates ratios of parameters for G_q_ that is bound and not bound to activated receptor. “+R” and “+A” label parameters for G_q_ bound to receptor or GAP.

Opening and closing of the nucleotide binding site is also reflected in the remarkably slow nucleotide association rates observed in the absence of receptor. The slow basal association rate constant for GTP, ∼500 M^−1^·s^−1^, is particularly striking, but all GDP and GTP association rate constants are less than 10^4^ M^−1^·s^−1^ without receptor stimulation. Receptor increases the association rates about 10^4^-fold to 10^6^–10^7^ M^–1^·s^−1^, values that are more commonly observed for binding of small ligands to proteins. Taken together with the slow rates of spontaneous nucleotide dissociation, the slow association rates indicate that the nucleotide binding site on G_q_ is essentially closed in the absence of receptor and that receptor stabilizes the open conformation regardless of whether GTP, GDP or no nucleotide is bound (see Discussion).

Second, comparison of the rate constants for nucleotide exchange shows that GAP potentiates the ability of the receptor to accelerate the dissociation of bound GDP by about 20-fold (Table 2). Thus, even though GAP has negligible effect on GDP binding by itself, its facilitation of GDP/GTP exchange helps minimize potential inhibition of signaling during stimulation by receptor. GAPs were not previously known to modulate GDP binding [1],[25], but this effect was probably overlooked because GAPs do not bind tightly to GDP-bound G protein; the RGAD complex will only be formed during steady-state GTPase turnover. GAP displays little effect on the rate of GTP dissociation because the binding of GAP and GTP to G protein is positively cooperative [1].

**Table 2:** Effect of GAP (A) on exchange catalyst activity of receptor (R)

Reaction	*k*_assoc_ (s^−1^)	+A/–A	*k*_dissoc_ (M^−1^·s^−1^)	+A/–A	*K*_eq_ (M^−1^)	+A/–A
t1	5.0×10^2^		2.2×10^−8^		2.3×10^10^	
t2 (+A)	3.8×10^2^	0.76	6.4×10^−10^	0.03	5.9×10^11^	26
t3 (+R)	1.7×10^6^		1.9×10^−2^		8.9×10^7^	
t4 (+A,+R)	2.1×10^6^	1.2	3.2×10^−2^	1.7	6.6×10^7^	0.73
d1	3.6×10^3^		1.5×10^−5^		2.4×10^8^	
d2 (+A)	6.8×10^3^	1.9	3.7×10^−5^	2.5	1.8×10^8^	0.77
d3 (+R)	2.1×10^7^		2.8×10^−1^		7.5×10^7^	
d4 (+A,+R)	1.5×10^6^	0.071	4.7	17	3.2×10^5^	0.004

Rate constants are taken from Table S2. “+A/–A” indicates ratios of parameters with and without saturating GAP (A). Note that GAP (A) increases the affinity of G_q_ for GTP and decreases the affinity for GDP.

The parameter set also indicates that receptor and GAP bind G protein negatively cooperatively, and that cooperativity depend on the binding of GDP or GTP (Table 3). Receptor and GAP reciprocally decrease the affinity of G_q_ for each other by 25-fold when GTP is bound and by ∼120-fold when GDP is bound, but there is essentially no cooperativity displayed for binding to nucleotide-free G_q_. The most striking result of this interaction is the rapid dissociation of GAP from the receptor-G_q_–GDP complex after GTP is hydrolyzed. The *t*_1/2_ for GAP dissociation is about 300 ms, about 90-fold faster than in the absence of receptor (Table S2). In contrast, GAP dissociation from GTP-bound G_q_ is slow, about 170-fold slower than hydrolysis, such that essentially every GAP binding event results in GTP hydrolysis. In summary, GAP dissociates virtually immediately after GTP hydrolysis during receptor-mediated signaling, and is thus potentially available to accelerate hydrolysis on other G proteins.

**Table 3:** Effect of receptor on affinity of G_q_ for GAP

GAP Binding to		*K*_eq_ (M^−1^)	+Rec/–Rec
G_q_		3.7×10^5^	
G_q_-GTP		9.6×10^6^	
G_q_-GDP		2.8×10^5^	
Rec-G_q_		5.6×10^5^	1.5
Rec-G_q_-GTP		3.9×10^5^	0.041
Rec-G_q_-GDP		2.3×10^3^	0.008

Values for *K*_eq_ are calculated from Table S2. Because the model is thermodynamically complete (Figure 1), effects of GAP on binding of receptor to G_q_ are identical to those shown here for effects of receptor on binding of GAP to G_q_.

**Figure 5:**
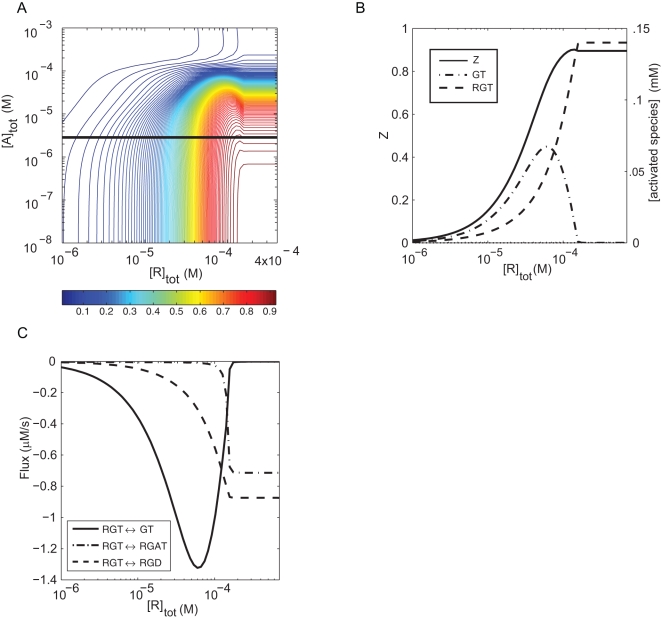
Steady-state activation of G_q_ under experimental conditions in reconstituted vesicles. (A) Fractional activation, *Z*, of 1.6×10^−4^ M total G_q_ was simulated at varying total concentrations of receptor and GAP using the rate constants in Table S2, 0.3 µM GTP and negligible GDP and P_i_. Note that molar concentrations of proteins are calculated in the annular volume of the membrane, but concentrations of nucleotides and other small molecules are calculated according to aqueous volume. (B) Steady-state concentrations of GTP-bound species (right axis) and *Z* (left axis) are plotted as functions of the total receptor concentration in the presence of 4.7×10^−6^ M GAP, indicated by the black line in (A). Concentrations of GAT and RGAT are not visibly different from zero. (C) Net fluxes (µM/s) for the reactions occurring after the branch-point species RGT are computed along the line shown in (A).

**Figure 6:**
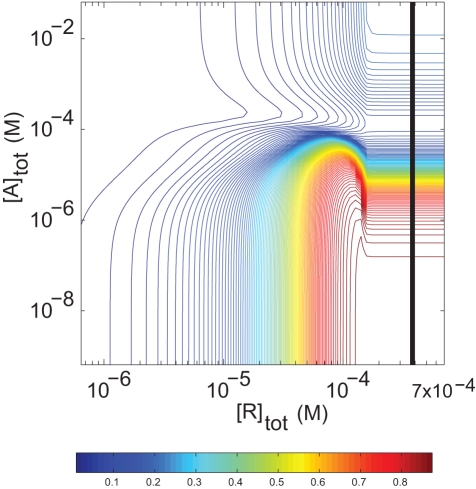
Steady-state activation of G_q_ under cellular conditions. Fractional activation *Z* was simulated at varying total concentrations of receptor and GAP, with 1.6×10^−4^ M G_q_, for cytosolic concentrations of 200 µM GTP, 20 µM GDP, and 1 mM P_i_. *Z* values for colors on the contour plot are defined in the reference bar below. Protein concentrations are expressed with reference to the membrane volume.

The nucleotide-dependent, negatively cooperative binding of receptor and GAP to G protein also helps determine the reaction pathway through the GTPase cycle: what intermediate species are populated and for how long (Figures 5 and 6; see below). For example, GTP accelerates the dissociation of receptor from G protein by ∼70-fold whereas GDP has a much smaller effect. This difference further biases receptor-promoted GDP/GTP exchange toward the forward (activating) direction. Qualitatively, destabilization of receptor binding by nucleotides confirms the observation that nucleotides drive dissociation of receptor from G protein [26].

### Coordinate Regulation of Signal Output by Receptor and GAP

To examine the overall regulatory behavior of the G protein module, we used the complete reaction model and average fitted parameter set to simulate signal output as the fraction *Z* of all G protein complexes to which GTP is bound. Figure 5A shows a contour plot of fractional activation at steady-state as a function of varying concentrations of receptor and GAP, using typical in vitro assay conditions to allow us to compare prediction with experiment (300 nM GTP, 10 pM GDP, no Pi). At low concentrations of active receptor, signal output is predictably low regardless of GAP concentration. In the absence of GAP (bottom of figure), increasing the concentration of receptor raises *Z* to about 93% activation. At saturating concentrations of GAP (top of figure), *Z* increases with increasing concentrations of active receptor to about 4% of maximal activation. This limiting value reflects the ratio of the rates of GTP hydrolysis to GDP/GTP exchange when GAP and receptor are both bound to G protein throughout the catalytic cycle. At high receptor concentration (right side), increasing concentrations of GAP causes *Z* to fall from 85% to 12%. These transitions are relatively smooth, although slopes are asymmetric and steeper than predicted by a Hill coefficient of 1. The values of *Z* at the corners agree with analytical calculations, which are only possible at these limits. While the precise output obviously depends on the values of the rate constants, the overall topography of this plot had sufficient similarity among fitted parameter sets to indicate that errors in the fit do not modify the essential behavior of the model.

The most striking feature of the *Z* contour plot lies in the region where the concentrations of G protein, receptor and GAP are similar. Here, *Z* contour lines are contorted and create an abrupt transition, a “ridge” at which activity peaks and then declines with increasing concentration of receptor. In a few locations, increasing the concentration of receptor causes *Z* to decrease, and in a tiny region, increasing the concentration of GAP actually increases *Z*. This unintuitive topography is not idiosyncratic to the average parameter set, but appears in various shapes for all the parameter sets obtained with repeats of the fitting procedure. To clarify the origin of this behavior, we calculated the fluxes and steady-state concentrations of intermediates at locations on either side of the ridge to determine what reactions and molecular species contribute to Z near the ridge (Figure 5C; see Figure S5 and Figure S6 for examples). To the left of the ridge, the major reaction path is RG→RGT→GT→GD→RGD→RG. GT is the major activated species. The receptor dissociates upon GTP binding and reassociates after hydrolysis, the mechanism referred to as collisional coupling [27]. GAP is not significantly involved in the reaction scheme and *Z* is low. Figure 5B indicates that the major active species is GT in this region. Across the ridge, the reaction pathway becomes a comparable mixture of RG→RGT→RGD→RG and RG→RGT→RGAT→RGAD→RGD→RG. Species RGT is the major active species (Figure 5A). Receptor remains bound throughout the GTPase cycle, and significantly, GAP is recruited to the receptor–G protein complex during the GTP-bound phase (Table 1). *Z* has a higher value despite involvement of the GAP in net GTPase turnover. The ridge thus reflects the coincidence of the peak in the concentration of GT in a region where the concentration of RGT is increasing significantly (Figure 5B).

The change in pathway is governed by choice of the reaction that follows the branch-point species RGT (Figure 5A and 5C). With increasing concentration of receptor, net flux switches from RGT→GT to RGT→RGAT and RGT→RGD as the concentration of receptor crosses the ridge. The peak in activity reflects the transient accumulation of GT as the concentration of free R increases and drives GDP/GTP exchange but before it reaches the level at which GAP is recruited. Above the Z ridge, flux through the GTPase cycle is maintained entirely by complexes that include receptor; i.e., where receptor remains bound throughout the catalytic cycle.

The occurrence of a ridge in *Z* with increasing receptor concentration, rather than a monotonic increase, is caused by the negatively cooperative binding of receptor and GAP to G protein (described above). The importance of this mechanism is indicated by the location of the ridge in the R-A plane. It lies just to the left of the line [A]_tot_ = [G]_tot_−[R]_tot_, where the sum of the concentrations of total receptor and total GAP equals the concentration of total G protein. This straight line appears as a curve on log–log plots (Figure 5A). Negatively cooperative binding of receptor and GAP to G protein make accumulation of RG and GA species far more likely than accumulation of RGA species and thus causes the abrupt shift of pathway and consequent peak in G protein activation. The crest of *Z* is displaced from the line because the GTPase cycle is not at equilibrium under steady-state reaction condition.

### G Protein Activation under Cellular Conditions

We also used the model and parameter set to simulate G protein activation under typical cytoplasmic conditions—0.2 mM GTP, 0.02 mM GDP, 1 mM P_i_ [28] (Figure 6). Activation of G_q_ responds to receptor and GAP in a pattern generally similar to that seen under laboratory assay conditions, but the higher cytoplasmic concentration of GTP allows substantial activation by receptor at high GAP concentrations. Signal output is thus significant, *Z*∼0.25, even in the presence of saturating GAP. Output remains high in the presence of GAP because GTPase flux is almost entirely from the RGA–>RGAT–>RGAD–>RGA pathway over a large part of the R-A plane (Figure S6, Figure S7, and Text S3). Given this pathway, high values of *Z* result in part from the GAP's potentiation of receptor-promoted GDP release (Table S2). GAP exerts this effect under cytoplasmic conditions because, at 0.2 mM GTP, nucleotide-free G protein binds GTP quickly (*t*_1/2_<2 ms) and because GAP does not dissociate appreciably. Equally important, receptor remains bound because GTP is hydrolyzed rapidly, before appreciable receptor can dissociate, and therefore catalyzes GDP/GTP exchange promptly after hydrolysis. The principal potentiating effect of cytoplasmic GTP concentration is thus to support continued association of receptor, GAP and G protein during the GTPase cycle.

A novel and unintuitive result of this simulation is the decline and subsequent increase in *Z* at high receptor concentrations as the concentration of GAP is increased. As shown in Figure 6, *Z* is minimal at about 10^−4^ M GAP and increases at higher GAP concentrations. This effect is not predicted for lower concentrations of GTP and is relatively small for the conditions and parameters used here. The occurrence and extent of this behavior depends sensitively on multiple rate constants, as do the relative plateau values of *Z* at high and low GAP concentration. In general, the ability of GAP to increase fractional G protein activation at high concentrations depends on its potentiation of the receptor's exchange catalyst activity and its indirect stabilization of receptor binding to G protein, as discussed above. Its mechanism is discussed in the Text S3.

### GAP Promotes Fast G Protein Deactivation upon Signal Termination

**Figure 7:**
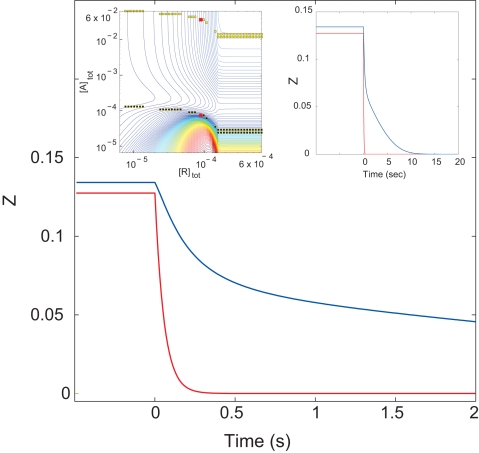
GAP-promoted deactivation of G_q_ after removal of receptor. Left inset: Segment of Figure 6 (cytosolic nucleotide concentrations) showing the results of a search of R-A space for pairs of high and low GAP concentrations where a 500-fold increase in GAP concentration caused less than 15% decrease of *Z* at steady-state but caused at least a 2-fold increase in the rate of deactivation when the concentration of receptor was instantaneously set to zero (see Materials and Methods). Dot spacing reflects the search grid. Pairs that met the criteria were found for all concentrations of receptor. G_q_ activation was first simulated at steady-state in the presence of 8.9×10^−5^ M receptor (red dots in left inset). At zero time, the concentration of receptor was set to zero. The upper and lower curves represent 7.4×10^−5^ M and 3.7×10^−2^ M GAP. Right inset shows the same deactivation reaction over a longer time.

In cells, GAP activity often accelerates signal termination when agonist is removed but does not inhibit signaling significantly while agonist is present [1]. To determine whether this behavior is accurately predicted by the present model and to study its mechanism, we simulated signal termination upon removal of a rapidly dissociating agonist by first allowing the system to reach steady state and then instantaneously setting the concentration of activated receptor to zero (Materials and Methods). We first scanned the receptor and GAP concentrations shown in Figure 6 for regions where increasing the GAP concentration causes minimal inhibition but significantly accelerates signal termination. Quantitative search criteria were chosen to mimic published experiments (reviewed in [1]; see legend to Figure 7), but their exact values are not crucial (results not shown). As shown in the inset to Figure 7, addition of GAP can accelerate deactivation with minimal steady-state inhibition at all concentrations of active receptor. A wide range of initial and final GAP concentrations also meet the initial criteria. This behavior is thus robust to initial conditions. Within this region, addition of GAP can accelerate signal termination up to 180-fold, which actually exceeds the acceleration that has been observed in cells.

Figure 7 shows the deactivation time course for a representative simulation that compares signal termination at high and low concentrations of GAP, shown as red dots in the inset. The higher GAP concentration accelerated G_q_ deactivation more than 15-fold, measured as time to 50% of initial activity, but inhibited receptor-stimulated G protein activation by only 5%. Qualitatively similar behaviors are observed over much of the area of Figure 6, indicating that fast termination combined with minimal inhibition is a common outcome of G protein GAP activity.

Neither termination time course in Figure 7 is monoexponential, and complete deactivation is markedly delayed at the lower GAP concentration (right inset). Some GAP activity thus appears to be required for reasonably fast decay of signal output to basal levels. Simulations with intermediate GAP concentrations (not shown) indicate that GAPs can also facilitate return to basal activation without accelerating signal termination to the extent shown in Figure 7, and a variety of termination behaviors can be observed at different points on this activation surface. While multiphasic decay of G protein signals has also been observed experimentally, we do not know whether the separate phases in Figure 7 correspond to specific cellular turn-off events.

Flux analysis of the deactivation events indicates that there is a single mechanism for accelerated signal termination by GAPs. At low GAP concentrations, the species RGT and RGAT both contribute significantly to activity in the presence of activated receptor. Upon removal of receptor, GT and GAT are rapidly created. GAT is then rapidly deactivated at a rate of 8.6 s^−1^ (*p*_21_ in Table S2), the initial phase of deactivation. The second, very slow phase is deactivation of GT. In contrast, at higher GAP concentrations almost all G protein activity is due to RGAT. When activated receptor is removed, the GAT that is formed hydrolyzes rapidly to cause fast deactivation. While deactivation is not really monophasic even at fairly high GAP concentrations, slow hydrolysis of GT is not significant because there is not much of it and because the GAP that dissociates from the GAD hydrolysis product binds remaining GT to accelerate its deactivation. In this way, GAP provides a pathway for fast signal turn-off without inhibiting signaling.

## Discussion

### Data-Constrained Modeling of a G Protein Signaling Module

A mechanistic model of signal transduction should provide quantitative understanding of how time-dependent outputs arise from the underlying binding, conformational and chemical reactions. This study attempts to address three unresolved mechanistic questions in G protein signaling. First, what are the underlying dynamics of the GTPase catalytic cycle that integrate the regulatory activities of receptors and GAPs, their reversible binding to the G protein, and their control of G protein activation? Which effects are important and what functions do they serve? Next, how can a GAP accelerate signal turn-off when agonist is removed, yet not inhibit activation while agonist is present? Both these questions are vital to understanding how G protein-regulated effectors such as phospholipase C-β and p115RhoGEF can act as GAPs for their G protein activators without blocking their own activation. Last, can we use a data-constrained model to quantitate the interactions and activities of multiple interacting proteins during steady-state signaling where one-by-one measurements are not feasible?

Quantitative modeling and simulation can provide this kind of understanding, but only if the underlying physical model is adequate and if the parameters of the mathematical model are objectively derived from experimental data. Even a relatively small G protein signaling module is a complex, non-linear system in which reaction pathways and modes of regulation may be both unintuitive and resistant to the simplifying assumptions of classical enzyme kinetics. We used a thermodynamically complete model, in which all reactions are reversible and all states are connected (Figure 1). Such a model assures that relevant reactions are not omitted, assures compliance with the laws of thermodynamics and uses detailed balance to help constrain parameters during the fitting process.

The present version of the model does omit two relevant reactions. First, the concentration of agonist-bound active receptor is used as a surrogate for the agonist-induced activation of a fixed number of receptors. This simplification precludes some pharmacological inferences, but no currently available mechanism quantitatively and accurately relates agonist binding, receptor activation and G protein regulation [29],[30]. Second, we omitted activation-induced dissociation of Gα_q_ and Gβγ. G protein subunits can dissociate in detergent solution [4], but physical dissociation in membranes is not universal [6],[31]. The binding of Gα_q_ to Gβγ in detergent solution suggests that dissociation is slower than the reactions studied here [11],[32], and preliminary data on fluorescence resonance energy transfer between Gα_q_ and Gβγ in phospholipid vesicles indicate that binding is relatively tight even for GTPγS-activated G_q_ (C. Hoang and E.M. Ross, unpublished). Thus, while certain behaviors determined here for G_q_ may reflect actions of both Gα_q_ and Gβγ subunits, kinetically significant dissociation is probably not an important factor. We also did not consider any direct effects of Gβγ on the actions of receptor or GAP because they are subsumed in the rate constants for the reactions of these multi-protein species. For example, it is plausible that Gβγ contributes to the stable association of receptor with GTP-bound Gα during rapid GTPase turnover, but we have no independent evidence for this effect.

Values for the rate constants for the model were derived from fits to steady-state GTPase data obtained with known concentrations of proteins, over widely varied concentrations of GAP, GTP and GDP, and in the presence or absence of agonist. Activities and ligand concentrations spanned several orders of magnitude. Such a dataset is appropriate for parameterizing a model of this complexity because steady-state activities encompass all the simultaneous reactions that modulate flux through the catalytic cycle, including those that cannot be measured individually. Indeed, most of the parameters could not have been determined by individual rate measurements regardless of desired accuracy or precision. We did not include pre-steady-state kinetic data in the fitting process, but individual rate constants that were previously directly determined in quenched flow experiments [12] agree well with those obtained here (Figure 3). The Metropolis-Monte Carlo fitting procedure yields a family of parameter sets that, with repetition, provides mean parameter values with quantitative statistical measures of accuracy. Most of the parameters also passed two other tests for validity: they were reproduced well in multiple fits to data (Figure 2) and, in fits to synthetic data, the fitted values reproduced the target values well (Figure 4). Further, thermal ensemble analysis indicated that the model was not significantly over- or under-parameterized (Figure S4 and Text S2). Thus, the data were sufficient in quality, quantity and diversity to produce reliable values for most of the rate constants. While the error windows on several of the parameters are larger than what would be expected from typical pre-steady-state measurement of a single enzymatic reaction rate, many are excellent even by traditional standards. The analysis also points out what parameters were not fit well, which prevents overinterpretation. For many of the poorly fit parameters, the chemical reactions do not take place to a significant extent, and their rates therefore do not contribute appreciably to steady-state GTPase activity or to G protein activation. Thus, they do not impact on interpretation of reaction rates or allosteric interactions, nor do they invalidate model-based simulations. Comparison of this parameter set with that of Bornheimer et al. shows several disagreements in values of reasonably well fit parameters for GTP and GDP binding in addition to expected disagreement with poorly fit values. Several are important for interpretation of allosteric interactions. Those authors chose their parameter set based on previously published pre-steady-state data from this laboratory, but did not fit them to a suitably diverse dataset. A significant value of the present fitting strategy is that it provides statistical descriptions of the reliability of individual rate constants, such that conclusions can be quantitatively evaluated. Having the complete set of rate constants allows simulation of signaling behavior with verifiable limits of accuracy.

This systems level kinetic analysis of G_q_ signaling provides three distinct but interrelated sets of mechanistic information. First, the fitting process provided values for previously inaccessible kinetic parameters and thus revealed novel cooperative interactions among receptor, G protein, GAP and nucleotides. Second, model-based simulation demonstrated how paths through the GTPase cycle vary with the concentrations and activities of the individual proteins. Third, these analyses combine to allow description of regimes where GAPs can facilitate rapid signal termination upon removal of agonist without substantially inhibiting signaling.

### Cooperative Interactions in G Protein Signaling

Because many of the important rate constants that describe the G protein signaling module were reasonably well determined by the fits to experimental data, this study identified several new regulatory interactions that control the rate and extent of G protein activation.

A major finding was that GAP potentiates the GDP/GTP exchange catalyst activity of the receptor (Table 2). GAP both accelerates GDP dissociation from the receptor-G protein complex and inhibits GDP rebinding, decreasing equilibrium affinity for GDP more than 200-fold. This effect of GAP contributes significantly to its ability to accelerate GTP hydrolysis without proportionately decreasing steady-state G protein activation by receptor. This effect could not be determined directly by standard pre-steady-state kinetics methods because it impacts only transient, low-affinity intermediates in the GTPase cycle. GAP had no significant effect on GDP binding in the absence of receptor, consistent with previous data [1], and had no significant effect on GTP binding to the receptor-G protein complex, although it increased the affinity for GTP of free G protein about 25-fold. This increase is consistent with the ability of GTP analogs to increase the affinity of G protein for GAPs [1]. Note that Gβγ contributes to the kinetics of nucleotide binding to Gα subunits and is intimately involved in its regulation by receptors [4] and GAPs [1],[23]. Our data do not distinguish the contributions of the individual subunits to the regulation of G_q_, but the net effects should represent the normal responses of intact G proteins in a biological membrane.

A second novel finding is that receptor significantly accelerates nucleotide binding to G protein in addition to promoting dissociation (Table 1). Fast GTP binding at cytosolic concentrations is crucial for maintaining high steady-state G protein activation (Figure 6). Acceleration of nucleotide binding also clarifies the mechanism of receptor-mediated nucleotide exchange. The receptor-promoted increase in the equilibrium *K*_d_ is much smaller than the increases in *k*_assoc_ and *k*_dissoc_ for both GTP and GDP (Table 1). The receptor acts thus primarily to open the nucleotide binding site, presumably by moving the switch regions away from the entrance, but does not drastically distort the binding site itself. Such movement is demanded by the structure of the Gα subunit because bound nucleotide is essentially covered by a protein lid in the closed conformation [33]. The intrinsic high affinity of G protein for GDP that derives from the covered site is crucial to maintain low basal activation in the absence of agonist-bound receptor. The site-opening mechanism described here allows the receptor to act as a highly efficient GDP/GTP exchange catalyst while maintaining adequate affinity of receptor for the nucleotide-bound forms of the G protein.

The idea that receptor opens the GTP binding site on G proteins actually dates to early studies of the GTPase cycle [34], but few studies have indicated that receptor actually increases *k*_assoc_ [35]–[37]. In contrast, the prototypical GTP-binding protein Ef-Tu is regulated primarily by negatively cooperative binding of the exchange factor Ef-Ts [38], and this is true for several other monomeric GTP-binding proteins and their exchange factors (GEFs) [39]–[41]. For these proteins, GDP dissociation is the primary regulated step and the increase in *k*_dissoc_ is roughly proportional to the increase in the equilibrium *K*_d_; effects on *k*_assoc_ are minimal. Negative cooperativity, defined as the reciprocal decrease in the equilibrium affinity of G protein for nucleotide and receptor when the other is present, is less significant for heterotrimeric G proteins than the ability of receptor to open the nucleotide binding site. Given the need for a low basal exchange rate, a purely negatively cooperative interaction with receptor would require a huge increase in *K*_d_ for GDP to allow receptor to promote physiologically fast exchange. The reciprocal effect on the *K*_d_ for receptor at physiological nucleotide concentrations would also compromise the stability of receptor binding. Heterotrimeric G proteins have thus evolved to use the lid of the binding site to allow low basal exchange without putting an energetically impractical demand on cooperative interaction with receptor.

The negative cooperative binding of receptor and GAP to G_q_ was also unexpected. This interaction could not readily be detected by conventional binding measurements because of the low affinity of GAPs for the GDP-bound form of G proteins (where negative cooperativity is greatest; see Table 3). It should now be possible to test this interaction directly using the parameter values found here to guide experimental design. Note that the reaction model (Figure 1) does not demand any direct or indirect interaction between receptor and GAP, and their negatively cooperative binding was shown by fitting to experimental data. The importance of this interaction is not intuitive, but it underlies the shape of the activation surfaces shown in Figures 5 and 6. Such a surface was also predicted by Bornheimer et al. [42], who based their model on data from this laboratory. Kinzer-Ursem and Linderman [43] also described a biphasic effect of receptor based on sensitivity analysis of a model that focused on receptor function without consideration of GAP. Our analysis indicates that the ridge of maximal activation approximates the line at which the total concentrations of receptor plus GAP equal that of G protein, and this prediction can now be used to analyze other systems where these concentrations vary. Interaction between receptor and GAP also largely dictates the pathways of intermediary reactions through the GTPase cycle as functions of the concentrations of receptor and GAP, and thus contribute to the transient kinetics displayed when agonist is either added or removed.

### Transient Responses and Signaling Dynamics

Simulations based on the parameterized model suggest mechanisms for how GAP activity promotes fast deactivation when agonist is removed without attenuating the signal while agonist is present. Receptor-generated signal output at steady-state can be significant over a wide range of GAP concentrations sufficient to accelerate signal turn-off (Figure 7). Such apparently paradoxical behavior is often observed for G protein-gated ion channels, whose cellular activation and deactivation kinetics can be studied directly [44],[45], reviewed in [1].

A major reason that a GAP can exert these two functions is its potentiation of the exchange-catalyst activity of the receptor, which is apparent by examining the rate constants that govern the GTPase cycle (Table 2). A second mechanism, which is evident only upon examining GTPase fluxes under the appropriate conditions, is that the GAP's multiple activities shift the path through the GTPase cycle such that receptor largely remains bound to G protein throughout the catalytic cycle and thus obviates the relatively slow step of reassociation with GDP-bound G protein after hydrolysis (Figure S5, Figure S6, and Figure S7). Thus receptor can initiate GDP/GTP exchange immediately after hydrolysis. Several properties of the GTPase reaction contribute to this effect, but it primarily results from the simple fact that GAP-stimulated GTP hydrolysis is faster than the rate of dissociation of receptor from the GTP-activated G protein. Because GDP dissociates faster than receptor, GDP dissociation occurs first and is followed by rapid GTP binding because the receptor maintains the nucleotide binding site in the open configuration. We refer to this mechanism as “kinetic scaffolding”, the ability of the GAP to promote long-term receptor binding by accelerating alternative reactions. We proposed this phenomenon previously [1],[11],[20], although we assumed that GAP also remains bound. The present analysis suggests that GAP binding to receptor-G-GDP is in rapid equilibrium, with dissociation likely to occur during each pass through the GTPase cycle. Because the affinity of G protein for GAP is poorly determined by these data (Figure 3), real quantitation of GAP binding is imprecise at best. Receptor binding is also not defined precisely in the fits to the present dataset, but examination of activation contours of the sort shown in Figure 6 show similar, although hardly identical, patterns when based on each of the 41 fitted parameter sets. The overall pattern of transit through the GTPase cycle is thus robust to variation in binding affinities over a reasonable range. Kinetic scaffolding was also proposed by Zhong et al. [16] based on nucleotide exchange kinetics. Kinetic scaffolding does not suggest any direct interaction, physical or allosteric, between receptor and GAP, but describes *functional and temporal* stabilization of receptor binding because alternative paths for receptor-G protein complex occur faster than dissociation. Kinetic scaffolding does not minimize the role of physical scaffolds, which can stabilize signaling complexes prior to activation by agonist (reviewed in [46]), and protein scaffolds may in some cases obviate the need for kinetic scaffolding. Kinetic scaffolding becomes efficient during signal transduction, however, by maintaining signaling proteins in their active complex. Further, kinetic scaffolding maintains receptor and G protein in contact and correctly oriented, whereas physical scaffolds may provide loose tethers which may be less effective.

Examination of the activation contour shows that deactivation upon removal of receptor (or, in cells, of agonist) is accelerated by GAP over a large and biologically important region of receptor-GAP space (Figure 7, inset). Deactivation is to some extent multiphasic at all points because activated species to which GAP is bound deactivate most rapidly, and further relatively fast deactivation depends on binding of GAP to other GTP-bound, activated species (Figure 7). Precise pathways vary depending on the concentration of GAP and fractional activation at the time receptor is removed. It is likely that such multiphasic deactivation occurs in cells upon removal of agonist, but determining the precise shape of such deactivation time courses is experimentally taxing, and determining the molecular events underlying each phase is not yet experimentally approachable. However, we can now use simulations of the sort shown in Figure 7 as guides to designing experimental studies of deactivation pathways.

Using computational modeling to analyze a specific dataset is valuable in that conclusions are based on real data and are statistically verifiable. However, the conclusions are to some extent unique to the particular proteins used in the experiments, and the experimental system used here is clearly simplified in comparison to the natural plasma membrane. However, a biochemically defined experimental system of intermediate size, such as this one, allows studies of complex regulatory interactions and their mechanisms that would be impossible in a plasma membrane where local protein concentrations are unknown and where effects of other components are difficult to rule out. It will be important to analyze other G proteins, effectors and GAPs in this way, both to determine important differences among G protein modules at the mechanistic level and to verify that this approach is generally valid. The details of agonist interactions with receptors in the context of a functioning signaling module is also of enormous interest, but there is insufficient understanding of these phenomena to incorporate them into a thermodynamically complete, data-driven model. Our approach is in this sense complementary to the rigorous but mechanistically speculative work of the sort pioneered by Linderman and coworkers [43],[47],[48]; see also [49]. We also need to engage questions of how GAPs function as effectors, and the present work will both guide these experiments and motivate direct measurements of the key interactions discovered so far.

## Materials and Methods

### Steady-State GTPase Assays

Steady-state GTPase activity was measured in large, unilamellar phospholipid vesicles that contain purified m1 muscarinic cholinergic receptor, Gα_q_β1γ2 and phospholipase C-β1 [11]. Vesicles were prepared as described and phospholipase was added subsequently. The average diameter of the vesicles is 71 nm diameter (SD = 5 nm) according to negatively stained electron microscopic images. Concentrations of each protein and the amount of recovered lipid were measured as described previously [11],[12]. For modeling, protein concentrations are calculated according to the volume of the vesicle bilayer (see below), which is itself calculated according to the concentration of total phospholipids in the vesicle suspension [11] and their averaged partial specific volume. Because the phospholipid bilayer is homogeneous, the concentration of each protein in each vesicle is assumed to be uniform. Vesicles contain an average of 0.8 to 5 receptors and 2 to 12 G_q_ molecules depending on their concentrations, which probably approximates their molar ratios in natural membranes [11]. The specific activity of agonist/GAP-stimulated GTPase activity in these vesicles varied by 37% (SD) among six preparations prepared over several months.

GTPase activity was assayed as described [11],[50]. The assay times and the amounts of vesicles used were adjusted to maintain steady-state activity high enough for reliable determinations. Specific activities were calculated according to receptor-accessible G_q_ in cases where agonist stimulation was measured [11]. Activity with no input from receptor was determined either in the presence of atropine, an inverse agonist, or in vesicles that did not contain receptors. Receptor-free vesicles probably displayed slightly lower activity than receptor-replete vesicles assayed with atropine, although the difference was uncertain because of difficulty in quantitating total Gα_q_ [22]. The GTPase datasets used in parameterization of the model are listed in (Table S1). In each, the concentration of one component (GTP, GDP, GAP) was varied while others were held constant. When the concentration of GTP is listed as equal to its *K*_m_, the value of *K*_m_ was determined under that set of assay conditions. The concentration of receptor varied among vesicle preparations, but was not itself varied systematically.

### Model Implementation

The biochemical model is implemented as a system of 14 ordinary differential equations that describe the concentrations of each of the protein species shown in Figure 1, plus free receptor and GAP (Figure S1). Concentrations of free GTP, GDP, and P_i_ are constants (*i.e.*, steady-state conditions) for the modeling and simulation reported here. There are 48 kinetic constants, labeled as shown in Figure 1.

Concentrations of receptor, G protein and GAP are calculated according to the volume of the lipid bilayer of the vesicles, and the total volume available for all proteins in the system is therefore the sum of the bilayer volumes of all the vesicles in the suspension [51]. This convention yields both second-order association rate constants and equilibrium association constants for protein-protein binding that are about 13,000-fold higher than would be calculated if concentration were expressed as the total aqueous assay buffer volume. First-order dissociation rate constants are not altered by this convention (Text S1). Proteins are assumed to be homogeneously distributed among all vesicles, and any local variation in concentration are assumed to be negligible. Specifically, the number of vesicles with one or more of the proteins absent is assumed to be negligible. Concentration of GTP, GDP, and P_i_ are calculated according to the aqueous assay volume.

### Global Fitting of Model Parameters to Experimental Data

To assign values to the kinetic constants appropriate to the m1 muscarinic receptor-G_q_-phospholipase C-β1 system, we simultaneously fitted all parameters listed in Figure S1 to all the data of the experiments in Table S1. Fitting minimizes the cost function, the total mean square deviation between the predictions of the model (*v*_mod_) and the data (*v*_data_), adjusted for the standard deviation (*σ*) of triplicate determinations.
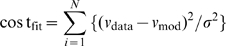
(1)To search parameter space, we used simulated annealing, an iterative stochastic search of multi-parameter space guided by the Metropolis algorithm [52],[53] (and references therein). At each iteration, the model is numerically integrated to yield steady-state GTPase activities and the cost function is calculated. Parameters are then changed randomly and the model is re-evaluated. Changes that decrease the cost function are accepted. Changes that increase the cost function may also be accepted, but only probabilistically according the Boltzmann probability function that depends on the cost difference of the proposed change scaled by an order parameter analogous to temperature in statistical physics. Simulated annealing applies the Metropolis algorithm while decreasing the temperature control parameter. The process allows escape from local minima of the cost manifold and discovery of the global minimum [53].

A thermodynamically complete model, with all possible interactions of species included and all reactions considered to be reversible, allows the use of thermodynamic constraints during the fitting process in addition to adjusting parameters to minimize the cost function. These constraints include both the path-independence of Δ*G* for reactions connecting two species (Δ*G* = 0 for any closed loop) and the net Δ*G* of hydrolysis of GTP to GDP and P_i_ that is enzyme-independent. In most fits, the parameter set was adjusted to meet thermodynamic constraints at each cycle of the search. Alternatively, thermodynamic constraints may be used quantitatively as part of the cost function. Deferring imposition of strict thermodynamic constraints may potentially allow broader, more ergodic search of parameter space during fitting, and this strategy was also evaluated.

In searches strictly constrained by path independence, each newly generated candidate parameter set was adjusted before recalculation of the cost function. Parameters to be recalculated to comply with path independence were chosen at random. A symbolic manipulator (Mathematica, Wolfram Research, Inc., Champaign, IL) was used to derive explicit expressions for all the possible combinations of recomputed parameter sets in terms of randomly generated ones. The subset of parameters to be recalculated was then chosen. This approach is valid because the constraint equations effectively reduce the number of independent kinetic parameters (degrees of freedom) in the system.

When strict constraints were deferred, each new, randomly generated parameter set was used whether or not it satisfied thermodynamics constraints to increase the potential ergodicity of the algorithm. In order to remediate this violation, a penalty term based on stoichiometric network theory (SNT) [54] was added to the cost function for the fit shown in Equation 1. SNT provides a method to compute sums of the chemical potential drops over each of the elemental loops *I* of the reaction network [54]. These target sums, shown as *s*_i_ for loop *i* in Equation 2, may be zero or non-zero depending on whether a particular loop includes a non-zero chemical motive force (hydrolysis of GTP). The penalty term expresses the weighted effect of deviation from the target values for all the elemental loops of the network. Its addition to the cost function thus causes the simulated annealing process to drive the fit toward simultaneously satisfying the thermodynamics constraints and minimizing the least-squares fit to the data. Overall, fits using SNT penalties were found to be comparable to fits using strict thermodynamic constraints, although SNT-constrained searches converged less rapidly. Ending SNT-constrained searches with a strict thermodynamically constrained search was an efficient way to combine both methods.
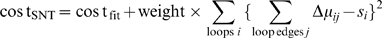
(2)


The system of coupled differential equations (Figure S1) was solved using the ode15s solver, which is designed for stiff systems of ordinary differential equations (Matlab, The MathWorks, Natick, MA). For efficiency, Matlab source code was automatically translated to C and compiled as a UNIX executable. The process was maximally parallelized because each data point can be calculated independently. A typical run employed 80 to 100 processors. Most runs were performed on the UNIX clusters of the Texas Advanced Computing Center, Austin, TX. Model-based simulations were also generated using ode15s, values of the kinetic constants shown in Table S2 and concentrations of proteins, nucleotides and P_i_ given in the text. Simulations were run to steady-state unless shorter times are specified. The integrity of numerical computations was verified throughout by checking for conservation of molecular types and by agreement with analytical solutions in limiting regimes where possible.

Each independent fitting search settles on a different parameter set which equivalently fits the data. Variability among fit results is due to the intrinsic coupling between parameters and the stochastic nature of the fit. We have verified that distributions of the logarithms of the association and dissociation constants from multiple search repeats are all peaked, unimodal and thus well approximated by single Gaussians. We derived a best estimate for each model parameter from the means of their logarithms. Similarly, we derived a measure of error on each fit parameter from the standard deviations of these distributions. This procedure is justified because the logarithm of a rate constant is proportional to activation energy; the average of logarithms preserves the validity of the thermodynamic relationships among them.

### Impulse Response

To simulate the response of G protein signaling to addition and removal of agonist, we first brought the system to an initial steady-state without receptor. We then instantaneously introduced a finite amount of activated receptor and allowed the model to reach a new steady-state. After 200 s, activated receptor was instantaneously removed and the system was allowed to return to the original steady-state. To reveal the mechanisms underlying the observed dynamics, the fractional activity *Z*, the fluxes and the concentrations of all species were computed as a function of time. Figure 7 shows a typical simulated output pulse shape (*Z* as function of time) and the reaction pathways responsible for it. We also surveyed the response to a pulse over a grid of receptor and GAP concentrations (2,500 grid points). At each point on the grid, we computed the time required for fractional activity to drop to *Z*_Max_/*e* where *Z*_Max_ is the plateau level of signaling output. To study mechanisms of GAP-accelerated signal termination under conditions where GAP minimally inhibits receptor-stimulated signaling, we searched the grid for locations where increasing the GAP concentration approximately 500-fold inhibited output by ≤5%. Locations where the higher GAP concentration accelerated signal at least two-fold are shown on inset of Figure 7. The mechanisms underlying the dynamic response were studied at selected points (Results).

## Supporting Information

Text S1 Reaction volumes and second order rate constants.
(0.02 MB DOC)

Text S2 Determining the quality of the fit using thermal ensembles.
(0.03 MB DOC)

Text S3 Interactive regulation by receptor and GAP under cytosolic conditions.
(0.03 MB DOC)

Table S1 GTPase assays used for fitting the model.
(0.03 MB DOC)

Table S2 Values of parameters.
(0.07 MB DOC)

Figure S1 Differential equations used to model the reactions of the GTPase cycle shown in Figure 1 of the main text.
(1.61 MB EPS)

Figure S2 Cost minimization during simulated annealing to fit parameters of the GTPase model under path-independence constraints (Materials and Methods). The parameters are fit to eight sets of GTPase data (Figure 2, Table S1). Blue symbols denote accepted moves; others are red. The green triangle shows the best fit to the data. Initial points are off-scale.
(1.40 MB EPS)

Figure S3 Two-stage cost minimization. The initial stage used non-conformance to stoichiometric network theory as a penalty (Materials and Methods). Strict path independence was enforced following step 2312 (dashed line). The small cost offset between the two minimization methods has been removed from the left part of the graph. Blue triangles show solutions accepted by the minimization algorithm; red dots are rejected solutions.
(2.87 MB EPS)

Figure S4 Analysis of the thermal ensemble of kinetic parameters about the best fit to data. (A) Fractional projections of eigenvectors of the covariance matrix onto the rate constants of the model are shown as colors on the calibration bar. Eigenvectors are ordered left to right from large eigenvalues to small eigenvalues (floppy to stiff). Eigenvectors 1 to 35 describe the degrees of freedom in the fit; the remaining 13 describe the thermodynamic constraints. (B) Forward and reverse kinetic parameters for each reaction are plotted according to projections of the 48 eigenvectors on each parameter. Projections of constraint eigenvectors are shown in red to highlight the forward to reverse indeterminacy innate to a thermodynamically constrained fit. For projections with absolute value >0.25, points are labeled with the eigenvector number and reaction name. Higher values of projections indicate that parameters are more independently measurable. Higher eigenvector numbers (1 to 35) indicate greater stiffness.
(3.03 MB EPS)

Figure S5 GTPase cycle fluxes determine fractional activation during steady-state turnover. Net fluxes (A) and unidirectional fluxes (B) through the GTPase cycle are shown for an intermediate GAP concentration (6.4×10^−6^ M) at 4×10^−4^ M receptor, the vertical line on Figure 6, at cytosolic nucleotide concentrations. Lengths of the arrows denotes relative flux. Where no arrows are visible, flux is not distinguishable from zero. Note that RGAT is the central intermediate for all utilized pathways. See Figure S6 for details.
(0.67 MB EPS)

Figure S6 The reaction path through the GTPase cycle changes with increasing concentrations of GAP. Net steady-state fluxes (forward minus reverse) for the two branch point reactions of RGT are shown as functions of the total concentration of GAP along the vertical line in Figure 6, which corresponds to 4×10^−4^ M receptor and cytosolic nucleotide concentrations. Fluxes are calculated from RGT and are therefore shown with negative values. On this scale, flux of RGT to GT is not distinguishable from zero (nor is GTP dissociation). At higher concentrations of GAP, flux from RGT approaches zero because RGT is no longer formed at a significant concentration. Total flux through the GTPase cycle ranges from 10 µM/s in the absence of GAP to 55×10^−5^ µM/s at saturating GAP.
(0.79 MB EPS)

Figure S7 Accumulation of individual activated G_q_ species changes with increasing concentrations of GAP. The steady-state concentration of each activated (GTP-bound) species (right axis) and net fractional G_q_ activation *Z* (left axis) are plotted as functions of the total concentration of GAP along the vertical line in Figure 6. The concentrations of GT and GAT are not distinguishable from zero on this scale.
(0.88 MB EPS)
