# Supplementary material for: Coordinate Regulation of G Protein Signaling via Dynamic Interactions of Receptor and GAP
Source: PLoS Comput Biol. 2008 Aug 15;4(8):e1000148. doi: 10.1371/journal.pcbi.1000148 (PMC2518520; doi:10.1371/journal.pcbi.1000148)
Supplement: Text S1 — Reaction volumes and second order rate constants. (0.02 MB DOC) [file pcbi.1000148.s001.doc]

Supporting Text S1.

Reaction volumes and second order rate constants

G protein, receptor, and GAP interact only within the annular volume of the vesicle surface and do not exchange among vesicles within the time span of the experiments. Their reaction volume is thus proportional to the ratio of their amounts to the amount of bilayer-forming lipid. It does not change as vesicles are diluted in aqueous suspension to alter the total GTPase activity measured in a particular experiment. We therefore calculated the concentrations of these proteins in terms of the annular volume of lipid, which was itself calculated according to the concentration of total lipid, published values for lateral lipid packing and an assumed annular shell thickness large enough to accommodate surface proteins [11,51]. Second-order association rate constants for protein-protein binding are also expressed in terms of this assumed reaction volume. See Runnells and Scarlata [51] for further discussion. Membrane-bound proteins also display three fewer diffusional degrees of freedom (one translational, two rotational), but the reaction model does not explicitly consider diffusion and these changes are subsumed in the values of the association rate constants.

Reaction volumes for binding of GTP and GDP are taken as the total aqueous reaction volume, and their association rate constants are calculated in these terms. These association rates can thus be compared with those for binding of other small molecules to proteins or with a calculated diffusional collision rate. For most high-activity experiments with low amounts of vesicles, the ratio of the aqueous volume to the annular membrane volume was 13,000.
