# Supplementary material for: Coordinate Regulation of G Protein Signaling via Dynamic Interactions of Receptor and GAP
Source: PLoS Comput Biol. 2008 Aug 15;4(8):e1000148. doi: 10.1371/journal.pcbi.1000148 (PMC2518520; doi:10.1371/journal.pcbi.1000148)
Supplement: Text S2 — Determining the quality of the fit using thermal ensembles. (0.03 MB DOC) [file pcbi.1000148.s002.doc]

Supporting Text S2.

Determining the Quality of the Fit Using Thermal Ensembles

Thermal ensemble analysis of the cost manifold near a minimum

For a complex system and a non-exhaustive data set, the optimally fit parameter set will consist of well-determined, less well-determined and, possibly, essentially undetermined parameters. Further, some parameters may be correlated, such that any individual parameter within a correlated group is not itself determined; fluctuation in one such parameter will force compensatory changes in others in the group. The shape of the cost function manifold in the neighborhood of the optimal solution encodes these correlations. To evaluate correlation of parameters, we used thermal ensemble analysis [24], a statistical sampling method that assesses the effect of nonlinearities in the neighborhood of the optimal solution. This analysis evaluates the dependence of the fit on groups of possibly correlated parameters. For thermal ensemble analysis, a large number of candidate parameter sets, the ensemble, is generated about the optimal set using the fit’s likelihood function, which upon assuming Gaussian independent errors, simplifies to a Boltzmann probability density function. Members of the ensemble contain parameters that give statistically equally valid fits to the data. Possible correlation of parameters reflect the shape of the cost manifold, which is encoded in , the matrix of parameter covariances. Use of the covariance matrix rather than the more difficult to compute Hessian matrix is discussed by Brown and Sethna [24].

Thermal ensemble analysis consists of an eigendecomposition of matrix  by singular value decomposition of the members of the ensemble [56]. The eigenvectors of  are ordered according to the size of their associated eigenvalues. Small eigenvalues denote stiff directions (large curvature, thus better defined) and the eigenvectors with large eigenvalues are soft directions (small curvature, thus less well defined). In addition, projections of the eigenvectors onto the model parameter vectors are measures of overlap of the model with the measurement process. In an ideal case, in which experiments measure kinetic parameters directly rather than as a complex output such as steady-state GTPase activity, the eigenvectors of  would be perfectly aligned with the underlying model (the projection would be unity along every parameter of the model). The projection allows identification of which parameters are mixed, whether they mix positively or negatively and the extent to which the combinations account for the data and alter the fit.

Results

The thermal analysis was performed to better understand how the fit parameters are coupled by the model, the measurement process and the available data. We used the log average of 41 independent searches (Fig. 3, Supp. Table 2) as the best estimate of the true parameters underlying the dynamics.

The thermal ensemble of parameter sets was generated randomly in the neighborhood of the best-fit parameter set according to a Boltzmann probability density function that matches the fit’s likelihood function. Distributions for all parameters were unimodal and show well-defined peaks centered on the fitted value (not shown). The eigendecomposition of the covariance matrix was done using the logarithms of the ensemble parameters [24] to account for the difference in scale between association and dissociation parameters and to stress how fractional parameter changes are coupled by the fit.

Supp. Fig. 4A shows how the eigenvectors of the covariance matrix of the thermal ensemble for the parameter set in Supp. Table 2 project upon individual kinetic parameters, a measure of how well the eigendirections align with the parameter set. The measurement process is not fully aligned with the model because eigenvectors project along more than one parameter. Some eigenvectors project along many parameters, but these projections are relatively small. Lack of generalized mixing suggests (1) a reasonable match between the model and the underlying phenomena , (2) the absence of severe over- or under-parameterization and (3) the availability of sufficient data. The eigenvectors in Supp. Fig. 4A are ordered left to right according to decreasing eigenvalues. Large eigenvalues indicate soft directions and correspond to a flatter manifold shape; small eigenvalues define stiff directions and correspond to a more curved manifold shape [24]. Eigenvectors 1 to 35 describe the degrees of freedom in the fit and range from soft to stiffer. Eigenvectors beyond 35 describe the 13 necessary and sufficient thermodynamic constraints (Methods), and are notably more stiff.

Supp. Fig. 4 B shows how eigenvectors project on forward and reverse kinetic parameters for individual reactions, and emphasizes that the constraint eigenvectors systematically project in equal and opposite directions onto forward and reverse kinetic parameter pairs. These projections clearly stand out on the diagonal with negative unit slope. Such indeterminacy is innate to the constrained fit process because thermodynamics constrains only the ratio in the pairs, not the individual parameter values. Many eigenvectors that are not simply thermodynamic constraints are generally better aligned with parameter pairs. Projections on better aligned parameter pairs, those with absolute value > 0.25, are individually labeled. Better aligned parameter pairs located in the lower left and upper right quadrants, away from the indeterminacy, may be well determined independently. Some of these parameters are relatively stiff (*e.g.* d3) while others are reasonably are soft (*e.g*. a6), depending on the available data. Parameter pairs for some other reactions are less well individually determined, those in the upper left and lower right quadrants. Irrespective of alignment, the softer eigenvectors of Supp. Fig. 4A show that all GAP kinetic parameters less well supplied in data. Similarly, the stiffer directions show that all the GDP parameters are much better supplied. These observations are corroborated by the relative size of these parameter error bars as shown on Fig. 3: GAP kinetic parameters have bigger errors than GDP kinetic parameters.

Overall, the results of the thermal analysis are consistent with the observed behavior of the fitting process. Although fits reach stable minima, mixing of parameters (reflected in the shape of the cost function around minima) allows more than one steady-state solution parameter set to fit the data, but the analysis of thermal ensembles points out coupled parameters. We repeated the thermal analysis using Monte Carlo-generated data and obtained similar results . For Monte Carlo, however, no reasonably well aligned parameter displayed innate indeterminacy owing to the more ideal nature of synthetic data (data not shown).

In summary, thermal analysis shows lack of generalized parameter mixing suggesting (1) a reasonable match between the model and the underlying phenomena, (2) the absence of severe over- or under-parameterization and (3) the availability of sufficient data for accurate determination of many of the parameters.

Reference

56. Joliffe, IT (2002) Principal Component Analysis. New York: Springer Verlag.
