# Supplementary material for: Coordinate Regulation of G Protein Signaling via Dynamic Interactions of Receptor and GAP
Source: PLoS Comput Biol. 2008 Aug 15;4(8):e1000148. doi: 10.1371/journal.pcbi.1000148 (PMC2518520; doi:10.1371/journal.pcbi.1000148)
Supplement: Text S3 — Interactive regulation by receptor and GAP under cytosolic conditions. (0.03 MB DOC) [file pcbi.1000148.s003.doc]

Supporting Text S3.

Interactive regulation by receptor and GAP under cytosolic conditions.

To understand how the concentrations of GAP and receptor determine the reaction path of the GTPase cycle for cytosolic nucleotide concentrations ((Fig. 6), we used the best-fit parameterization of the model (Supp. Table 2) to compute the steady-state concentration of each intermediate and both net and elemental (unidirectional) fluxes for each reaction. As an example, we consider reactions and species along the vertical line in Fig. 6 (4x10^-4^ M receptor; membrane concentration) that describe how Z initially decreases to a minimum and then recovers significantly as the concentration of GAP is increased. This progression is qualitatively similar for all receptor concentrations above 8x10^-6^ M, or one receptor per 20 molecules of G_q_. This range thus includes concentrations found in most cell membranes. The relative values of Z at low, intermediate and high GAP concentrations depend on the precise values of key rate constants for GDP/GTP exchange and GTP hydrolysis, but the maintenance of substantial G_q_ activation at high concentrations of GAP is a general prediction of the model for cytosolic concentrations of GTP. Key fluxes and the concentrations of two GTP-activated species along this line are shown in Supporting Figs. 6 and 7.

At low GAP concentrations, net flux is carried through the RG-->RGT-->RGD-->RG path (see Fig. 1), with predictably high Z because the rate of hydrolysis of GTP in the RGT complex is significantly slower than the GDP/GTP exchange rate (Supp. Fig. 6). The only significant activated species is RGT (Supp. Fig. 7). At lower receptor concentrations (to the left of the line), GT and GD species also contribute to net flux through the catalytic cycle (Supp. Fig. 5), the classical collisional coupling pathway [27].

As the concentration of GAP is increased and Z begins to decline, the

RG--> RGT -->RGD-->RG pathway is essentially eliminated for two reasons. First, the linear RGA-->RGAT-->RGAD-->RGA pathway becomes significant and carries about half of the net flux. Second, the RGT that is formed binds GAP to form RGAT faster than it hydrolyzes GTP (Supp. Fig. 6). The amount of RGAT remains small, however, because its hydrolytic rate is faster than the rates of the two reactions from which it is formed.

As the concentration of GAP is increased further and Z reaches its minimum, essentially all net flux is on the RGA-->RGAT-->RGAD-->RGA pathway, but GDP/GTP exchange remains slow because GAP tends to dissociate from RGAD. While RGAD and RGD are in rapid equilibrium, RGD predominates and its GDP dissociation rate is slower because GAP potentiates the exchange catalyst activity of the receptor in the RGAD complex. This situation is also shown graphically in Supp. Fig. 5.

At high GAP concentrations, Z again increases because the RGD <==> RGAD equilibrium shifts to RGAD and the exchange rate is therefore increased. Both GDP dissociation and GTP binding are maximally fast.
