# Supplementary material for: Coordinate Regulation of G Protein Signaling via Dynamic Interactions of Receptor and GAP
Source: PLoS Comput Biol. 2008 Aug 15;4(8):e1000148. doi: 10.1371/journal.pcbi.1000148 (PMC2518520; doi:10.1371/journal.pcbi.1000148)
Supplement: Table S1 — GTPase assays used for fitting the model. (0.03 MB DOC) [file pcbi.1000148.s004.doc]

Supporting Table 1. GTPase assays used for fitting the model.

**[Carbachol] [GTP] [GDP] [PLC-β1]**

10^-4^ var 0 2x10^-8^

10^-4^ K_m_ var 2x10^-8^

10^-4^ 2x10^-6^ 0 var

10^-4^ var 0 0

0 var 0 2x10^-8^

0 K_m_ var 2x10^-8^

0 var 0 0

0 K_m_ var 0

Steady-state GTPase assays used in fitting the model are described according to the reagents that were varied and those held constant. Data from these assays are shown in Fig. 2. Concentrations of receptor and G_q_ were held constant as described in the text. “var”: varied from zero to near saturation. “K_m_”: concentration equal to the experimentally determined K_m_ under the specified assay conditions. The aqueous phase concentration of PLC-β1 corresponds to approximately 2.5x10^-4^ M in the annular vesicle volume.
