# Supplementary material for: Coordinate Regulation of G Protein Signaling via Dynamic Interactions of Receptor and GAP
Source: PLoS Comput Biol. 2008 Aug 15;4(8):e1000148. doi: 10.1371/journal.pcbi.1000148 (PMC2518520; doi:10.1371/journal.pcbi.1000148)
Supplement: Table S2 — Values of parameters. (0.07 MB DOC) [file pcbi.1000148.s005.doc]

Supporting Table 2. Values of parameters

| Reaction | Rate Constant | σ _down_ | σ _up_ | Reaction | Rate Constant | σ _down_ | σ _up_ |
| --- | --- | --- | --- | --- | --- | --- | --- |
| a1,1 | 2.5 x 10 ^4^ | 2.4 x 10 ^4^ | 1.9 x 10 ^6^ | a1,2 | 6.7 x 10 ^-2^ | 6.6 x 10 ^-2^ | 3.1 |
| a2,1 | 2.5 x 10 ^5^ | 2.4 x 10 ^5^ | 9.5 x 10 ^6^ | a2,2 | 2.6 x 10 ^-2^ | 2.5 x 10 ^-2^ | 1.4 |
| a3,1 | 7.7 x 10 ^3^ | 7.5 x 10 ^3^ | 2.7 x 10 ^5^ | a3,2 | 2.8 x 10 ^-2^ | 2.8 x 10 ^-2^ | 4.5 |
| a4,1 | 2.8 x 10 ^3^ | 2.6 x 10 ^3^ | 5.7 x 10 ^4^ | a4,2 | 5.0 x 10 ^-3^ | 4.7 x 10 ^-3^ | 8.1 x 10 ^-2^ |
| a5,1 | 3.4 x 10 ^4^ | 3.3 x 10 ^4^ | 1.1 x 10 ^6^ | a5,2 | 8.7 x 10 ^-2^ | 8.3 x 10 ^-2^ | 2.1 |
| a6,1 | 5.4 x 10 ^3^ | 5.3 x 10 ^3^ | 2.9 x 10 ^5^ | a6,2 | 2.4 | 2.3 | 4.1 x 10 ^1^ |
|  |  |  |  |  |  |  |  |
| r1,1 | 1.4 x 10 ^7^ | 1.4 x 10 ^7^ | 5.2 x 10 ^8^ | r1,2 | 6.8 x 10 ^-4^ | 6.6 x 10 ^-4^ | 2.7 x 10 ^-2^ |
| r2,1 | 4.3 x 10 ^6^ | 4.0 x 10 ^6^ | 6.7 x 10 ^7^ | r2,2 | 5.0 x 10 ^-2^ | 4.7 x 10 ^-2^ | 7.7 x 10 ^-1^ |
| r3,1 | 1.3 x 10 ^7^ | 1.2 x 10 ^7^ | 6.2 x 10 ^8^ | r3,2 | 1.9 x 10 ^-3^ | 1.9 x 10 ^-3^ | 1.1 x 10 ^-1^ |
| r4,1 | 8.5 x 10 ^6^ | 8.0 x 10 ^6^ | 1.4 x 10 ^8^ | r4,2 | 2.7 x 10 ^-4^ | 2.6 x 10 ^-4^ | 7.6 x 10 ^-3^ |
| r5,1 | 4.0 x 10 ^5^ | 3.7 x 10 ^5^ | 7.0 x 10 ^6^ | r5,2 | 1.1 x 10 ^-1^ | 1.1 x 10 ^-1^ | 3.4 |
| r6,1 | 8.4 x 10 ^5^ | 8.1 x 10 ^5^ | 2.8 x 10 ^7^ | r6,2 | 1.6 x 10 ^-2^ | 1.5 x 10 ^-2^ | 1.0 |
|  |  |  |  |  |  |  |  |
| t1,1 | 4.9 x 10 ^2^ | 2.7 x 10 ^2^ | 6.1 x 10 ^2^ | t1,2 | 2.2 x 10 ^-8^ | 1.8 x 10 ^-8^ | 1.1 x 10 ^-7^ |
| t2,1 | 3.8 x 10 ^2^ | 2.0 x 10 ^2^ | 4.1 x 10 ^2^ | t2,2 | 6.4 x 10 ^-10^ | 5.8 x 10 ^-10^ | 6.3 x 10 ^-9^ |
| t3,1 | 1.7 x 10 ^6^ | 9.6 x 10 ^5^ | 2.2 x 10 ^6^ | t3,2 | 1.9 x 10 ^-2^ | 1.7 x 10 ^-2^ | 1.8 x 10 ^-1^ |
| t4,1 | 2.1 x 10 ^6^ | 3.0 x 10 ^5^ | 3.5 x 10 ^5^ | t4,2 | 3.1 x 10 ^-2^ | 2.9 x 10 ^-2^ | 3.6 x 10 ^-1^ |
|  |  |  |  |  |  |  |  |
| p1,1 | 1.8 x 10 ^-2^ | 1.7 x 10 ^-2^ | 2.1 x 10 ^-1^ | p1,2 | 5.1 x 10 ^-6^ | 4.1 x 10 ^-6^ | 2.2 x 10 ^-5^ |
| p2,1 | 1.8 x 10 ^1^ | 1.6 x 10 ^1^ | 2.4 x 10 ^2^ | p2,2 | 1.7 x 10 ^-1^ | 1.6 x 10 ^-1^ | 2.7 |
| p3,1 | 6.2 x 10 ^-3^ | 5.8 x 10 ^-3^ | 9.1 x 10 ^-2^ | p3,2 | 2.2 x 10 ^-8^ | 2.1 x 10 ^-8^ | 3.1 x 10 ^-7^ |
| p4,1 | 1.5 x 10 ^1^ | 1.2 x 10 ^1^ | 6.6 x 10 ^1^ | p4,2 | 9.5 x 10 ^-3^ | 9.0 x 10 ^-3^ | 1.7 x 10 ^-1^ |
|  |  |  |  |  |  |  |  |
| d1,1 | 3.6 x 10 ^3^ | 1.8 x 10 ^3^ | 3.7 x 10 ^3^ | d1,2 | 1.5 x 10 ^-5^ | 1.1 x 10 ^-5^ | 4.4 x 10 ^-5^ |
| d2,1 | 6.8 x 10 ^3^ | 3.0 x 10 ^3^ | 5.5 x 10 ^3^ | d2,2 | 3.7 x 10 ^-5^ | 2.7 x 10 ^-5^ | 9.9 x 10 ^-5^ |
| d3,1 | 2.1 x 10 ^7^ | 1.8 x 10 ^7^ | 1.0 x 10 ^8^ | d3,2 | 2.8 x 10 ^-1^ | 2.2 x 10 ^-1^ | 1.1 |
| d4,1 | 1.5 x 10 ^6^ | 6.0 x 10 ^5^ | 1.0 x 10 ^6^ | d4,2 | 4.7 | 1.8 | 2.9 |

Values of the rate constants that describe the GTPase reaction model (Fig. 1) were determined by fitting to steady-state data as described in Experimental Procedures. Values are log averages of 41 fitting runs (Fig. 3). Because log averages were used, equivalent to averaging ΔG^‡^,

asymmetric values of s.d. are shown as σ_up_ or σ_down_. Units are s^-1^ or M^-1^**^.^**s^-1^.
